# Identification of novel genes associated with exercise and calorie restriction effects in skeletal muscle

**DOI:** 10.18632/aging.204793

**Published:** 2023-06-12

**Authors:** Jae Sook Kang, Min Ju Kim, Eun-Soo Kwon, Kwang-Pyo Lee, Chuna Kim, Ki-Sun Kwon, Yong Ryoul Yang

**Affiliations:** 1 Aging Convergence Research Center, Korea Research Institute of Bioscience and Biotechnology (KRIBB), Daejeon 34141, Republic of Korea; 2 Department of Bimolecular Science, KRIBB School of Bioscience, Korea University of Science and Technology (UST), Daejeon 34113, Republic of Korea; 3 Aventi Inc., Daejeon 34141, Republic of Korea; 4 Department of Bioinformatics, KRIBB School of Bioscience, Korea University of Science and Technology (UST), Daejeon 34113, Republic of Korea

**Keywords:** exercise, calorie restriction, myogenesis, insulin sensitivity, mitochondrial respiration, muscle atrophy

## Abstract

Exercise and caloric restriction (CR) significantly increase longevity across a range of species and delay aging-related losses in organ function. Although both interventions enhance skeletal muscle function, the molecular mechanisms underlying these associations are unknown. We sought to identify genes regulated by CR and exercise in muscle, and investigate their relationship with muscle function. To do this, expression profiles of Gene Expression Omnibus datasets obtained from the muscle tissue of calorie-restricted male primates and young men post-exercise were analyzed. There were seven transcripts (*ADAMTS1*, *CPEB4*, *EGR2, IRS2*, *NR4A1*, *PYGO1*, and *ZBTB43*) that were consistently upregulated by both CR and exercise training. We used C2C12 murine myoblasts to investigate the effect of silencing these genes on myogenesis, mitochondrial respiration, autophagy, and insulin signaling, all of which are processes affected by CR and exercise. Our results show that in C2C12 cells, *Irs2* and *Nr4a1* expression were critical for myogenesis, and five genes (*Egr2*, *Irs2*, *Nr4a1*, *Pygo1*, and *ZBTB43*) regulated mitochondrial respiration while having no effect on autophagy. *Cpeb4* knockdown increased the expression of genes involved in muscle atrophy and induced myotube atrophy. These findings suggest new resources for studying the mechanisms underlying the beneficial effects of exercise and calorie restriction on skeletal muscle function and lifespan extension.

## INTRODUCTION

Aging is associated with many disorders, including myocardial infarction, stroke, cancers, macular degeneration, osteoarthritis, and neurodegenerative diseases. Several rejuvenation strategies that improve health and lifespan have recently emerged, such as use of blood factors, senescent cell ablation, and cellular reprogramming [1]. However, there are two issues with these strategies: many of their underlying biological mechanisms are unknown, and their use is impeded by the negative side-effects that some cause. By contrast, physical exercise and caloric restriction (CR) are safe, potent, non-pharmacological interventions that improve lifespan. In addition, both interventions improve metabolism and delay the onset of age-associated diseases including diabetes, brain atrophy, arthritis, cardiovascular disease, osteoporosis, and sarcopenia [2, 3].

Both CR and exercise improve muscle function, including regeneration capacity, insulin sensitivity, autophagy, and mitochondrial respiration. For example, muscle satellite cells are essential for muscle regeneration, but their function gradually diminishes during physiological and pathological aging; however, moderate exercise training enhances muscle regeneration after injury [4] since myokines produced during exercise play a role in muscle regeneration and satellite cell proliferation [5]. In addition, short-term CR increases the number of muscle stem cells and improves muscle regeneration [6].

Mitochondria are critical for maintaining skeletal muscle energy balance. Mitochondrial number and function are determined through adaptive reprogramming in response to a variety of physiologic or pathological stressors, including CR and exercise. In aged mice, CR maintains mitochondrial capacity and efficiency without increasing mitochondrial biogenesis [7]. Exercise is also regarded as a non-pharmacological method to preserve mitochondrial health due to the numerous intracellular pathways activated during exercise [8]. By contrast, mitochondrial dysfunction is linked with the development of skeletal muscle insulin resistance. In line with this, accumulating evidence shows that both exercise and CR improve insulin sensitivity in animals and humans [9–11].

In skeletal muscle, autophagy is a critical catabolic process crucial for substance turnover and energy production and consumption. Autophagic dysfunction in muscle causes cellular changes such as mitochondrial damage, decreased sarcomeric protein turnover, and cell death, which leads to the development of numerous skeletal muscle diseases. There is evidence that autophagy is induced in skeletal muscle in response to exercise, depending on its intensity and duration [12, 13]. In rodent skeletal muscle, moderate CR reduces the age-related decrease in autophagy [14].

In this study, we explored upregulated genes in Gene Expression Omnibus (GEO) datasets of skeletal muscle samples isolated from calorie-restricted primates and young men post-exercise [15, 16]. We discovered genes that were upregulated by both interventions, seven of which were selected for evaluation of their role in cellular function.

## RESULTS

### Identification of differentially expressed genes resulting from CR and exercise in muscle

**Figure 1:**
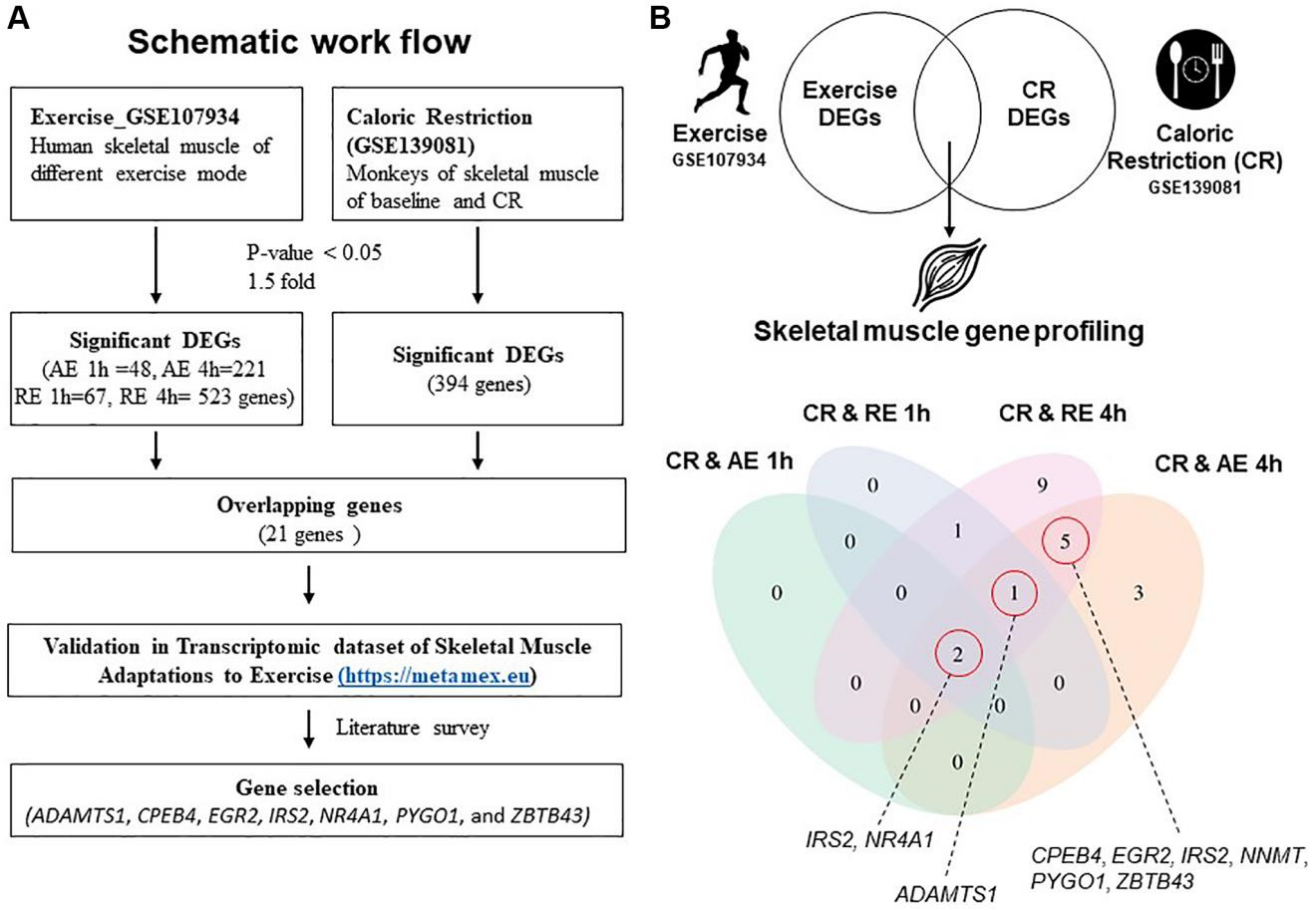
**Schematic workflow and gene profiling in skeletal muscle.** (**A**) Schematic workflow of analysis using GSE107934 and GSE139081 datasets. (**B**) Significantly regulated genes from exercise and CR presented in a Venn diagram. Abbreviations: AE: aerobic exercise; CR: caloric restriction; DEG: differentially expressed gene; RE: resistance exercise.

To identify genes involved in enhancing muscle function, we investigated upregulated genes in skeletal muscle specimens collected from calorie-restricted primates and young men post-exercise (Figure 1A). The dataset with NCBI-GEO accession GSE107934 (for physical exercise) and GSE139081 (for CR) were retrieved [15, 16]. GSE107934 data consist of sample files collected from skeletal muscle at 1 and 4 h post-aerobic (AE) and post-resistance exercise (RE) from young men (27 ± 3 years; *n* = 6) in a crossover study [16]. In this study, AE was performed for 40 minutes on a stationary bike at 70% maximal heart rate, while RE consisted of 8 sets of 10 repetitions at 65% of 1-repetition maximum (1RM). GSE139081 data was obtained from adult (27.8 ± 1.7 years; *n* = 5 for control and *n* = 8 for CR) male rhesus monkeys that had been part of the University of Wisconsin Aging and Caloric Restriction study for 18 years [15]. A flowchart of the analysis process for the GEO datasets is shown in Figure 1A.

Of the 394 genes changed by CR, 21 overlapped with genes changed by the two exercise modes and time points using pre-defined differentially expressed gene (DEG) results (Figure 1B). The number of shared DEGs increased at the later (4 h) post-exercise timepoint in both AE and RE modes: 11 and 18 genes overlapped with CR at 4 h AE and 4 h RE, respectively (Figure 1B, Table 1). We first selected two genes that were upregulated in all five groups (CR, AE 1 h, AE 4 h, RE 1 h, and CR4 h) of DEGs. Next, we identified one overlapping gene from four groups (CR, RE 1 h, RE 4 h, and AE 4 h), and five overlapping genes from three groups (CR, RE 4 h, and AE 4 h) (Figure 1B). Ultimately, eight genes were selected that overlapped between the DEGs induced by CR and those induced exercise; a disintegrin-like and metallopeptidase with thrombospondin type 1 motif 1 (*ADAMTS1*), cytoplasmic polyadenylation element binding protein 4 (*CPEB4*), early growth response 2 (*EGR2*), insulin receptor substrate 2 (*IRS2*), nicotinamide N-methyltransferase (*NNMT*), nuclear receptor subfamily 4, group A, member 1 (*NR4A1*), pygopus family PHD finger 1 (*PYGO1*), and zinc finger and BTB domain containing 43 (*ZBTB43*). Among these, *NNMT* gene is a significant DEG in both CR and exercise conditions, but it was excluded as a candidate gene because its expression decreases with CR and increases with exercise. Next, MetaMEx [17], a dataset of skeletal muscle transcriptional responses to different modes of exercise, was used to determine whether the selected genes from our analysis were also associated with exercise. In human skeletal muscle, acute exercise, but not chronic exercise, upregulated the selected genes (Supplementary Figure 1). Therefore, seven genes were ultimately selected (*ADAMTS1*, *CPEB4*, *EGR2*, *IRS2*, *NR4A1*, *PYGO1*, and *ZBTB43*).

**Table 1:** List of intersection genes of CR and exercise.

**Gene Name**	**Description**	**Log2 FC**	
**CR & AE 1 h**		**CR**	**AE 1 h**
IRS2	Insulin receptor substrate 2 [Source: HGNC Symbol; Acc: HGNC: 6126]	2.0167	1.552
NR4A1	Nuclear receptor subfamily 4 group A member 1 [Source: HGNC Symbol; Acc: HGNC: 7980]	2.8673	2.504
**CR & AE 4 h**		**CR**	**AE 4 h**
ADAMTS1	ADAM metallopeptidase with thrombospondin type 1 motif 1 [Source: HGNC Symbol; Acc: HGNC: 217]	1.962	1.956
ZBTB43	Zinc finger and BTB domain containing 43 [Source: HGNC Symbol; Acc: HGNC: 17908]	3.8696	0.998
ZBTB40	Zinc finger and BTB domain containing 40 [Source: HGNC Symbol; Acc: HGNC: 29045]	2.6263	−1.294
NNMT	Nicotinamide N-methyltransferase [Source: HGNC Symbol; Acc: HGNC: 7861]	−3.725	2.163
LINC01128	Long intergenic non-protein coding RNA 1128 [Source: HGNC Symbol; Acc: HGNC: 49377]	2.7968	1.032
PYGO1	Pygopus family PHD finger 1 [Source: HGNC Symbol; Acc: HGNC: 30256]	2.1175	1.177
LPIN1	Lipin 1 [Source: HGNC Symbol; Acc: HGNC: 13345]	2.4499	0.879
EGR2	Early growth response 2 [Source: HGNC Symbol; Acc: HGNC: 3239]	2.147	1.645
IRS2	Insulin receptor substrate 2 [Source: HGNC Symbol; Acc: HGNC: 6126]	2.0167	1.529
NR4A1	Nuclear receptor subfamily 4 group A member 1 [Source: HGNC Symbol; Acc: HGNC: 7980]	2.8673	2.364
CPEB4	Cytoplasmic polyadenylation element binding protein 4 [Source: HGNC Symbol; Acc: HGNC: 21747]	2.1907	0.959
**CR & RE 1 h**		**CR**	**RE 1 h**
ADAMTS1	ADAM metallopeptidase with thrombospondin type 1 motif 1 [Source: HGNC Symbol; Acc: HGNC: 217]	1.962	1.322
IER5	Immediate early response 5 [Source: HGNC Symbol; Acc: HGNC: 5393]	3.5284	1.938
IRS2	Insulin receptor substrate 2 [Source: HGNC Symbol; Acc: HGNC: 6126]	2.0167	1.55
NR4A1	Nuclear receptor subfamily 4 group A member 1 [Source: HGNC Symbol; Acc: HGNC: 7980]	2.8673	2.649
**CR & RE 4 h**		**CR**	**RE 4 h**
JOSD1	Josephin domain containing 1 [Source: HGNC Symbol; Acc: HGNC: 28953]	2.3048	1.378
ASXL1	ASXL transcriptional regulator 1 [Source: HGNC Symbol; Acc: HGNC: 18318]	3.0485	0.761
ARHGEF7	Rho guanine nucleotide exchange factor 7 [Source: HGNC Symbol; Acc: HGNC: 15607]	−2.1106	−0.778
ADAMTS1	ADAM metallopeptidase with thrombospondin type 1 motif 1 [Source: HGNC Symbol; Acc: HGNC: 217]	1.962	2.318
IER5	Immediate early response 5 [Source: HGNC Symbol; Acc: HGNC: 5393]	3.5284	1.939
ZBTB43	Zinc finger and BTB domain containing 43 [Source: HGNC Symbol; Acc: HGNC: 17908]	3.8696	1.105
ALOX5	Arachidonate 5-lipoxygenase [Source: HGNC Symbol; Acc: HGNC: 435]	3.254	1.481
NNMT	Nicotinamide N-methyltransferase [Source: HGNC Symbol; Acc: HGNC: 7861]	−3.725	2.361
FOSL1	FOS like 1, AP-1 transcription factor subunit [Source: HGNC Symbol; Acc: HGNC: 13718]	2.574	1.575
PYGO1	Pygopus family PHD finger 1 [Source: HGNC Symbol; Acc: HGNC: 30256]	2.1175	1.073
EGR2	Early growth response 2 [Source: HGNC Symbol; Acc: HGNC: 3239]	2.147	1.398
IDI1	Isopentenyl-diphosphate delta isomerase 1 [Source: HGNC Symbol; Acc: HGNC: 5387]	−2.307	1.077
ATF6	Activating transcription factor 6 [Source: HGNC Symbol; Acc: HGNC: 791]	2.3729	0.847
IRS2	Insulin receptor substrate 2 [Source: HGNC Symbol; Acc: HGNC: 6126]	2.0167	2.641
RASSF8	Ras association domain family member 8 [Source: HGNC Symbol; Acc: HGNC: 13232]	2.2496	0.987
NR4A1	Nuclear receptor subfamily 4 group A member 1 [Source: HGNC Symbol; Acc: HGNC: 7980]	2.8673	2.645
CPEB4	Cytoplasmic polyadenylation element binding protein 4 [Source: HGNC Symbol; Acc: HGNC: 21747]	2.1907	1.531
WIPF3	WAS/WASL interacting protein family member 3 [Source: HGNC Symbol; Acc: HGNC: 22004]	2.7017	1.106

### *Irs2* and *Nr4a1* are key genes in myogenesis

**Figure 2:**
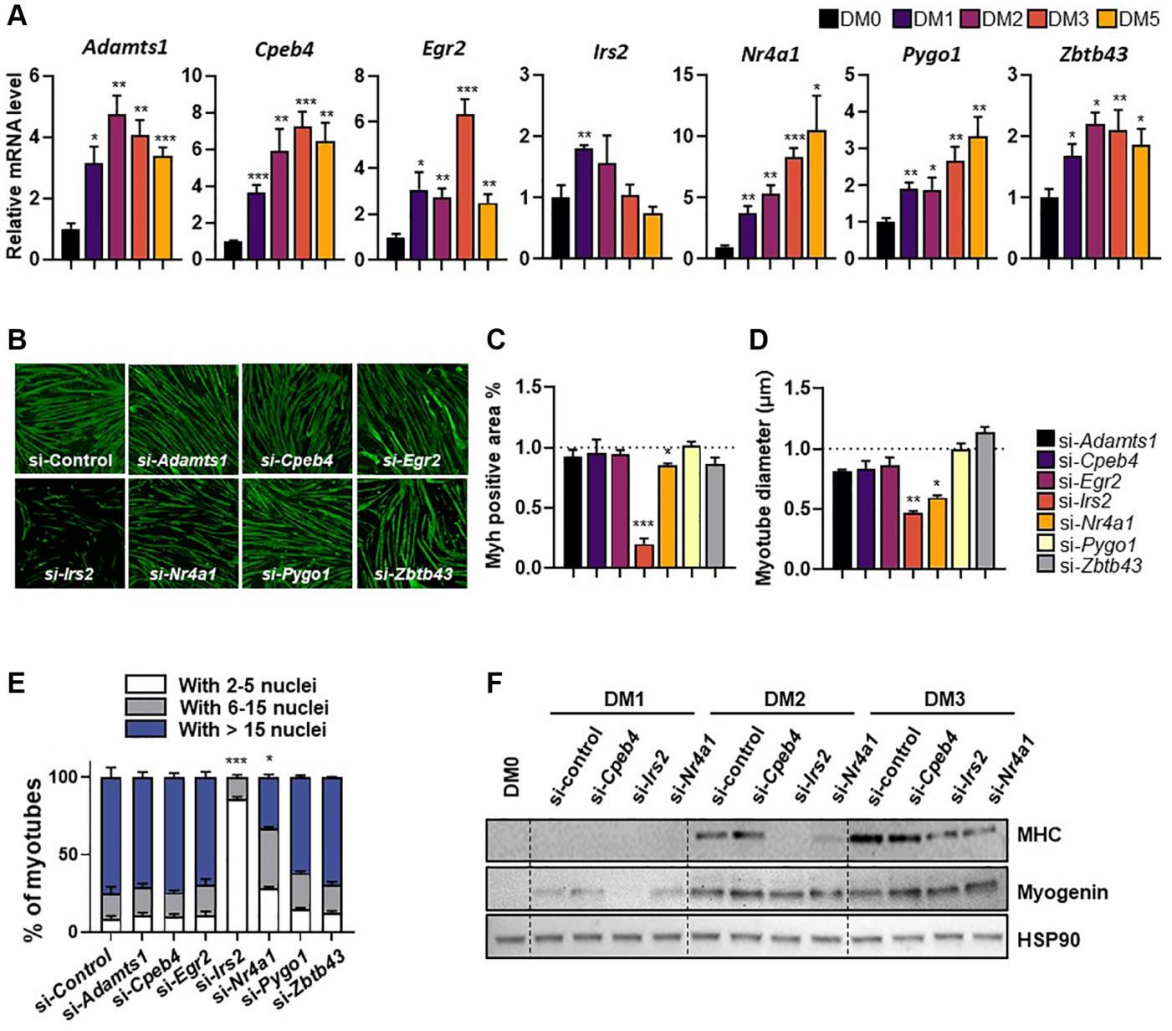
**Expression patterns of selected genes during differentiation of C2C12 myotubes.** (**A**) Relative gene expression of target genes in differentiating C2C12 cells at 0-, 1-, 2-, 3-, and 5-days post-induction. mRNA levels were normalized to *36b4* mRNA levels, as measured by qPCR analysis. The data are represented as mean ± SEM. Statistically significant differences are denoted as ^*^p < 0.05, ^**^p < 0.01, ^***^p < 0.005 vs. DM0. (**B**) Representative images of myosin heavy chain (MHC; green) immunostaining in myoblasts transfected with target genes and differentiated for 5 days. (**C**) Quantification of myotube myosin-positive (Myh+) areas, (**D**) myotube diameter, and (**E**) fusion index. (**F**) Western blot showing expression of MHC and myogenin, differentiation markers, during differentiation (0-, 1-, 2-, and 3-days). The data are represented as mean ± SEM. Statistically significant differences are denoted as ^*^p < 0.05, ^**^p < 0.01, ^***^p < 0.005 vs. si-*Con*.

CR and physical exercise improve muscle repair by modulating muscle satellite cell availability and activity [4, 6]. C2C12 murine myoblasts were used to investigate the role of the seven selected genes in myogenesis. Specifically, mRNA expression levels of the identified genes were measured during C2C12 myoblast differentiation using quantitative real-time PCR. Both *Nr4a1* and *Pygo1* mRNA expression exhibited a significant, time-dependent increase during C2C12 differentiation over 5 days; by contrast, *Adamts1*, *Cpeb4*, *Egr2*, *Irs2*, and *Zbtb43* expression increased initially (peaking at days 2–4), before declining (Figure 2A). To further investigate the involvement of these genes in myogenesis, C2C12 myoblasts were transfected with each siRNA one day before the induction of differentiation and differentiation was induced for 3 days. The efficiency of siRNA knockdown was confirmed by measuring mRNA levels of each gene 24 hours after transfection (Supplementary Figure 2). Then, we investigated the expression of myosin heavy chain (MHC), a marker of late differentiation in myogenesis, by immunofluorescence in C2C12 cells (Figure 2B). From this, the rate of myotube differentiation was estimated by quantifying myosin-positive areas and myotube diameters. Both *Nr4a1* and *Irs2* knockdown resulted in significantly lower MHC-positive areas and myotube diameters than control siRNA treatment (both *p* < 0.05; Figure 2C, 2D). A significantly lower myogenic fusion index was also observed in *Nr4a1* or *Irs2* knockdown cells (Figure 2E). Consistent with this, knockdown of *Nr4a1* and *Irs2* genes also resulted in lower MHC and myogenin protein levels than control siRNA treatment, whereas *Cpeb4* knockdown had no effect on the expression of these markers in C2C12 myotubes (Figure 2F). These data suggest that of the selected genes, expression of NR4A1 and IRS2 is required for myogenesis.

### Selected genes do not affect starvation-induced autophagy and glucose uptake

**Figure 3:**
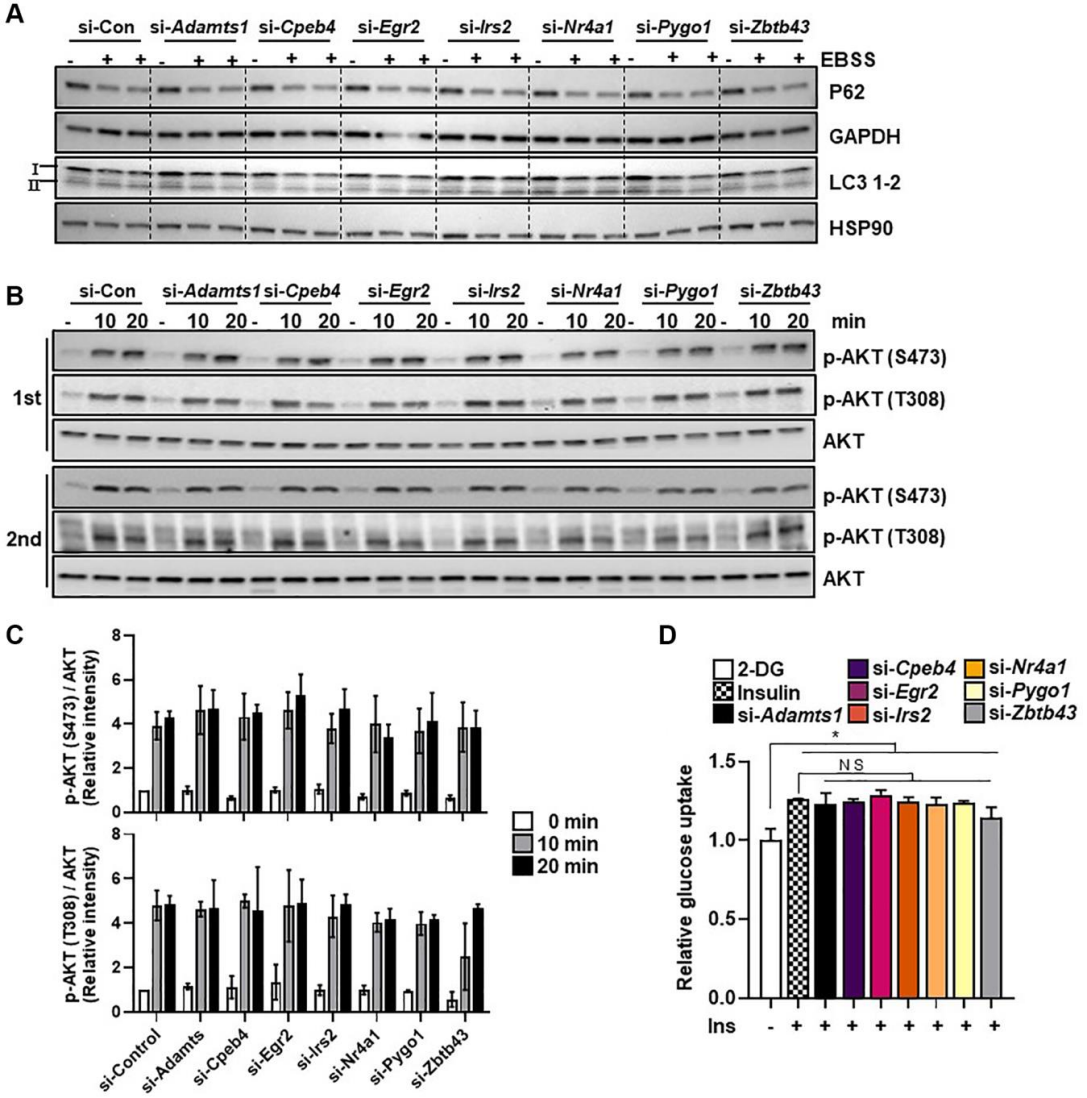
**Selected genes do not regulate starvation-induced autophagy.** (**A**) C2C12 myotube cells were transfected with selected genes or control siRNAs and exposed to EBSS for 6 h to induce autophagy. The autophagy markers LC3B-1/2 and P62 were measured in protein extracts using western blot analysis; GAPDH and HSP90 were used as loading controls. (**B**) The insulin signaling molecules p-AKT and AKT (**C**) were quantified using western blot analysis and densitometry (*n* = 3). (**D**) Glucose uptake in C2C12 myotubes transfected with selected genes (*n* = 3). All data are represented as mean ± SEM. Statistically significant differences are denoted as ^*^p < 0.05, ^**^p < 0.01, ^***^p < 0.005.

Since both exercise and CR enhance autophagy, which contributes to their beneficial effects on skeletal muscle health, we examined whether the selected genes were involved in autophagy regulation. Each of the selected genes were silenced in C2C12 myotubes and the cells were subjected to a 6-hour EBSS treatment, which is commonly used to induce autophagy. Immunoblotting was used to assess the levels of P62, LC3B-1, and LC3B-2 expression. Neither P62 degradation nor the conversion of LC3B-1 to LC3B-2 was altered by knockdown of the selected genes under starvation conditions in myotubes (Figure 3A), suggesting that these genes were unrelated to autophagy.

Exercise and CR are both linked with significant improvements in insulin sensitivity. We investigated the effect of gene silencing on insulin-induced AKT phosphorylation and glucose uptake in C2C12 myotubes. Compared with the control, neither glucose uptake nor AKT phosphorylation (T308 and S473) was affected by knockdown of each gene in C2C12 myotubes (Figure 3B–3D). These results suggest that the selected genes do not have a measurable role in autophagy and glucose uptake in myotubes.

### Selected genes affect mitochondrial respiration

**Figure 4:**
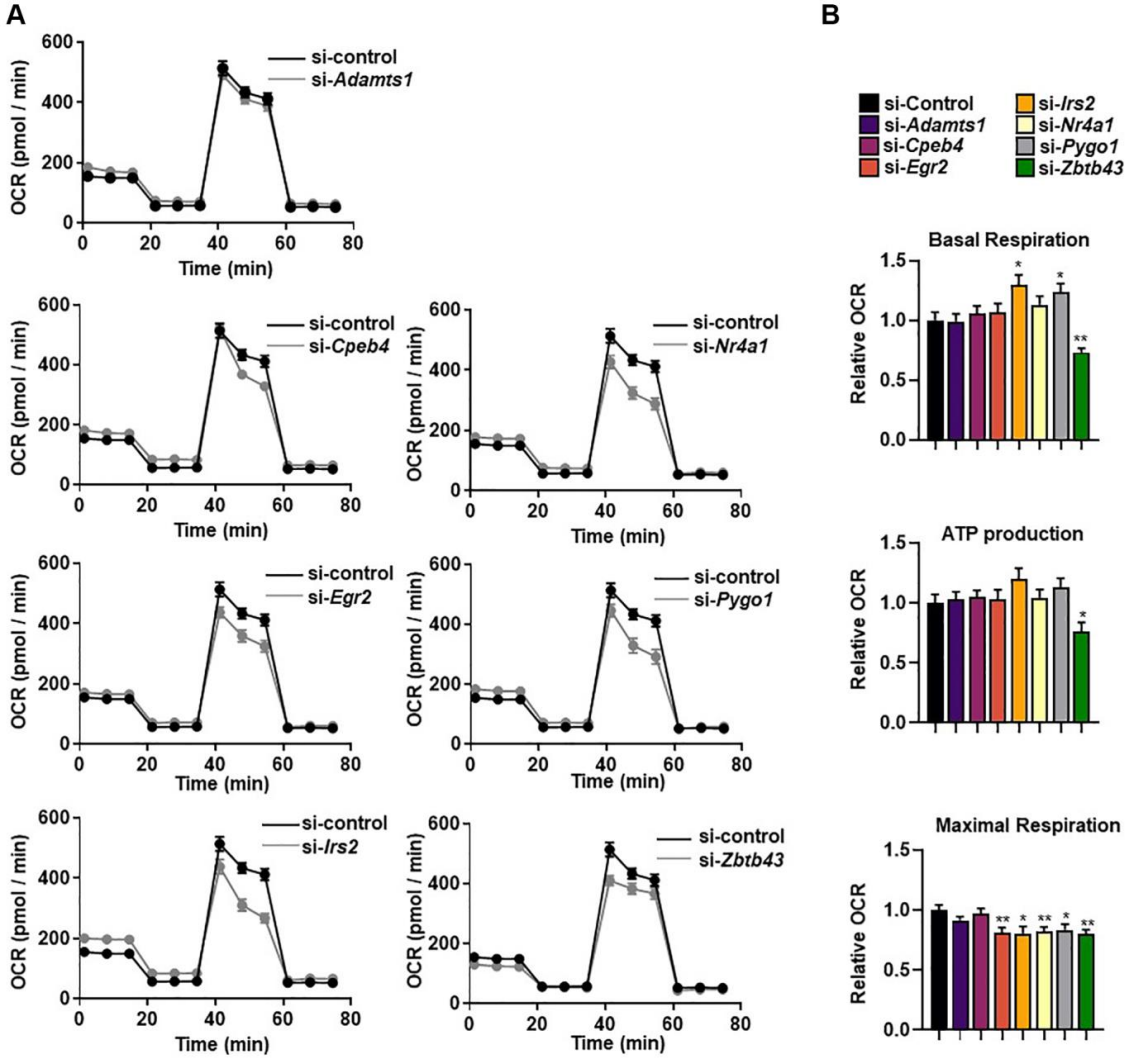
**Selected genes regulate mitochondrial respiration.** (**A**) The real-time oxygen consumption rate (OCR) of selected gene-transfected C2C12 myotubes was determined using a Seahorse XFe96 analyzer. (**B**) The area under the curve (AUC) values of basal respiration, ATP-linked respiration, and maximal mitochondrial respiration. OCRs were normalized to total cellular protein. All data are represented as mean ± SEM. Statistically significant differences are denoted as ^*^p < 0.05, ^**^p < 0.01, ^***^p < 0.005.

CR and exercise improve mitochondrial function, biogenesis, and bioenergetic efficiency [8, 18]. To confirm whether the selected genes were involved in regulation of mitochondrial respiration, we silenced the genes in C2C12 myotubes and evaluated mitochondrial function using bioenergetic parameters including basal respiration, maximum respiration, and ATP production-coupled oxygen consumption rate (OCR). Basal mitochondrial respiration was significantly higher after *Irs2* and *Pygo1* knockdown, and significantly lower after *Zbtb43* knockdown, than that in control siRNA-treated C2C12 myotubes (all *p* < 0.05; Figure 4B). Notably, knockdown of five genes (*Egr2*, *Irs2*, *Nr4a1*, *Pygo1*, and *ZBTB43*) resulted in significantly lower levels of FCCP-induced maximal respiration compared with those in control cells (all *p* < 0.05; Figure 4B). By contrast, there were no significant changes in ATP production-coupled respiration after gene knockdown (Figure 4A, 4B). These results suggest that *Egr2*, *Irs2*, *Nr4a1*, *Pygo1*, and *ZBTB43* genes are important for mitochondrial function in C2C12 myotubes.

**Figure 5:**
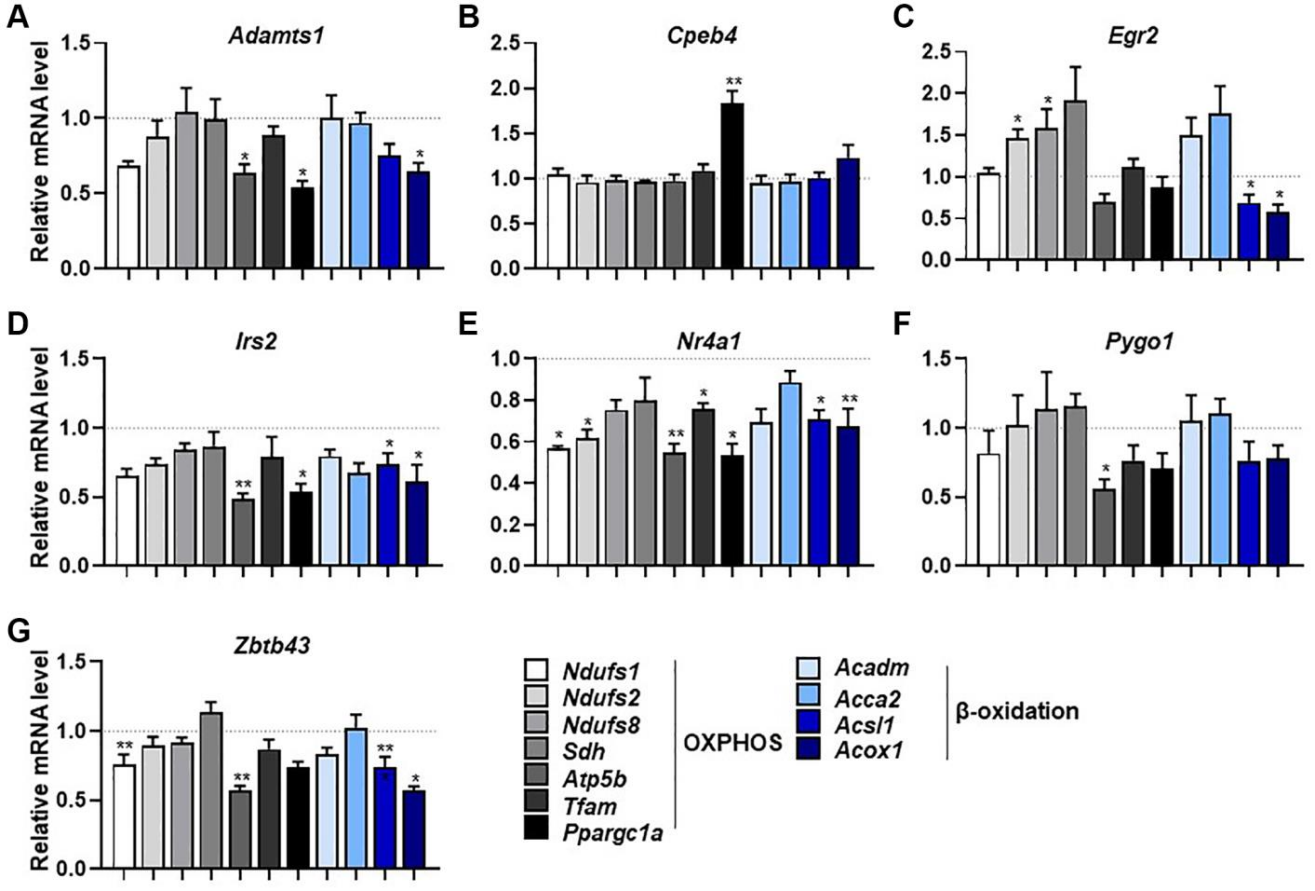
**Selected genes regulate mitochondrial respiration and β-oxidation-associated gene expression.** (**A**–**G**) mRNA expression of mitochondrial respiration-related genes (*Ndufs1, Ndufs2, Ndufs8, Sdh, Atp5b, Tfam,* and *Ppargc1a*) and β-oxidation-related genes (*Acadm, Acca2, Acsl1,* and *Acox1*) measured by qPCR in C2C12 myotubes with knockdown of selected genes (*n* = 3). All data are represented as mean ± SEM. Statistically significant differences are denoted as ^*^p < 0.05, ^**^p < 0.01, ^***^p < 0.005.

We next investigated how the selected genes influence the expression of mitochondrial respiration-related genes. The expression levels of several OXPHOS-related genes were significantly lower after knockdown of *Adamts1*, *Irs2*, *Nr4a1*, *Pygo1*, and *Zbtb43* genes compared with those in control cells (*p* < 0.05; Figure 5A–5G). However, *Egr2* knockdown resulted in significantly higher expression levels of some OXPHOS genes but lower levels of β-oxidation-related genes (Figure 5C). In addition, some β-oxidation-related genes were downregulated by knockdown of *Adamts1*, *Egr2*, *Irs2*, *Nr4a1*, and *Zbtb43,* but not by knockdown of *Cpeb4* and *Pygo1* (Figure 5A–5G). The mechanism in which these genes expression affects mitochondrial respiration has still not fully elucidated. Nonetheless, these findings imply that the selected genes function in mitochondrial respiration and β-oxidation in muscle.

### *Cpeb4* knockdown induces myotube atrophy via a catabolic pathway

**Figure 6:**
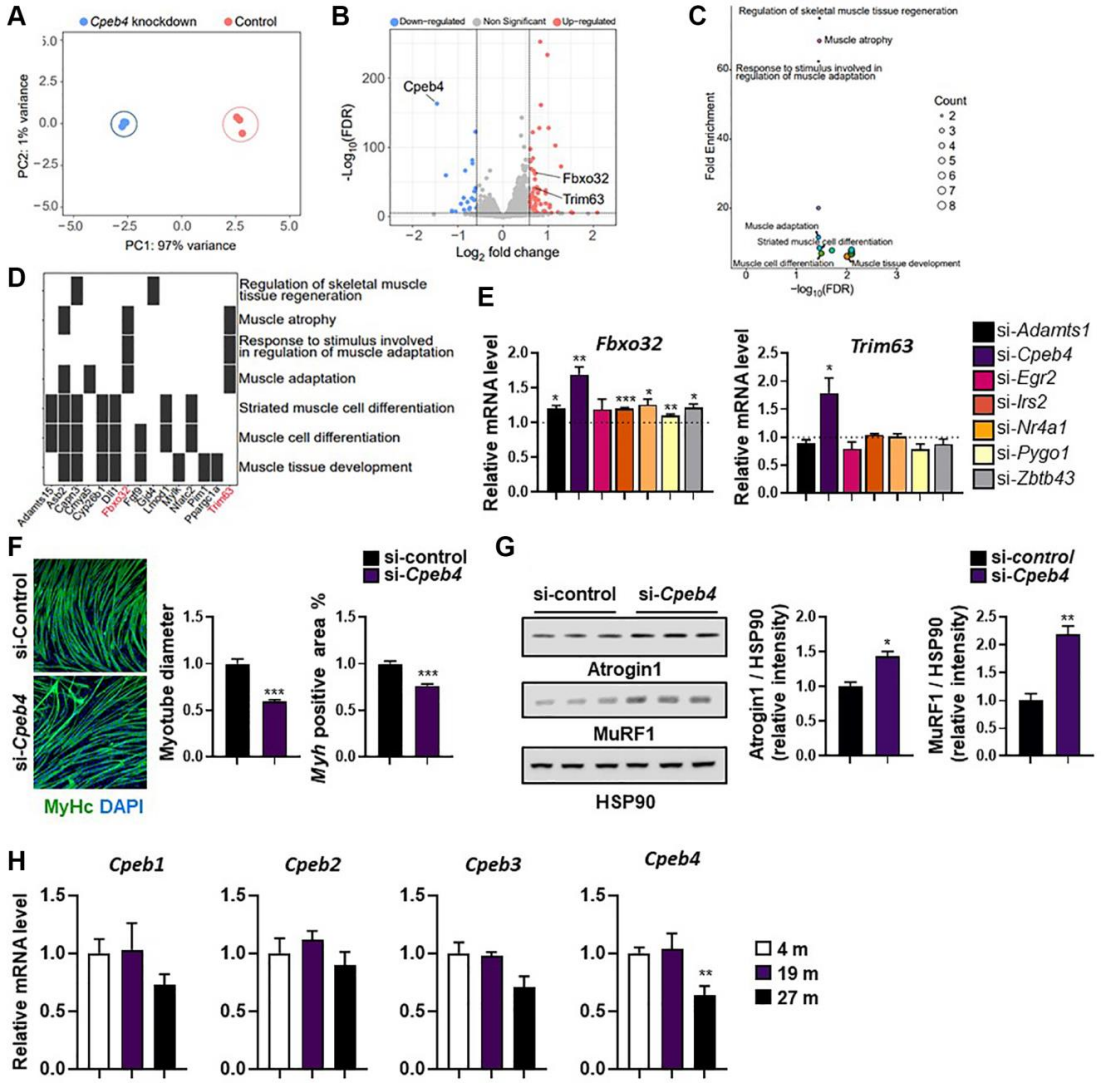
**Knockdown of *Cpeb4* induces atrophy in C2C12 myotubes.** (**A**) Principal component analysis (PCA) of RNA-seq datasets from C2C12 myotubes for control and *Cpeb4*-knockdown cells. (**B**) Volcano plot of differential expression analysis. (**C**) Summarized gene ontology terms related to biological processes. (**D**) Functional annotation clustering using Database for Annotation, Visualization, and Integrated Discovery. (**E**) mRNA expression of genes related to muscle wasting (*Fbxo32, Trim63*), measured by qPCR. (**F**) Representative image of myosin-stained *Cpeb4* knockdown (si-*Cpeb4*) or control siRNA-treated (si-control) myotubes differentiated for 5 days. Quantification of myotube diameter and myosin-positive (Myh+) area. (**G**) Western blot showing expression of atrophy-related protein levels (Atrogin-1 (*Fbxo32*) and MuRF-1 (*Trim63*)) and quantification of target protein expression relative to HSP90 using densitometry analysis (*n* = 3). (**H**) Relative mRNA level of different *Cpeb* isoforms (*Cpeb1–4*) in the tibialis anterior muscle in young (4-month-old) and aged mice (19- and 27-month-old; (*n* = 4, 5). All data are represented as mean ± SEM. Statistically significant differences are denoted as ^*^p < 0.05, ^**^p < 0.01, ^***^p < 0.005.

As reported above, *Cpeb4* knockdown had no impact on myogenesis, OCR, or the insulin signaling pathway. Although *Cpeb4* mRNA is expressed at high levels in muscle and is increased by exercise, its role in muscle is uncertain. To investigate the global transcriptome changes induced by *Cepb4* knockdown, we performed RNA-seq analysis on control and *Cpeb4*-knockdown myotubes. Principle component analysis revealed two distinct populations that were separated into control and *Cpeb4*-knockdown cells (Figure 6A). Compared with control cells, 115 DEGs (82 upregulated, 33 downregulated) were found in *Cpeb4*-knockdown C2C12 cells (Figure 6B); *Cpeb4* was the most downregulated gene in *Cpeb4*-knockdown cells, as expected. To examine which biological processes were affected by *Cpeb4* knockdown, we performed over-representation analysis using gene ontology (GO) terms. For upregulated genes after *Cpeb4* knockdown, seven of the 14 enriched GO terms were muscle-related gene sets, such as muscle atrophy (GO:0014889), muscle adaptation (GO:0043500), and regulation of skeletal muscle tissue regeneration (GO:0043416) (Figure 6C). In total, ~17% of upregulated genes were muscled function-related genes. Among the top upregulated genes after *Cpeb4* knockdown of myotubes were *Trim63* and *Fbxo32*, genes associated with atrophy (Figure 6D).

To confirm the RNA-seq results, we measured *Trim63* and *Fbxo32* by qPCR in C2C12 myotubes. Compared with control siRNA-treated C2C12 cells, knockdown of *Adamts1*, *Cpeb4*, *Irs2*, *Nr4a1*, *Pygo1*, and *Zbtb43* resulted in significant upregulation of *Fbxo32* expression (all *p* < 0.05; Figure 6E). *Trim63* mRNA significantly increased only when *Cpeb4* was silenced. In line with the RNA-seq results, both *Trim63* and *Fbxo32* mRNA expression were strongly upregulated by *Cpeb4* knockdown (both *p* < 0.05; Figure 6E). Therefore, we investigated whether *Cpeb4* expression might control the expression of *Trim63* (Murf-1) and *Fbxo32* (Atrogin-1) in myotubes and contribute to myotube atrophy. C2C12 cells were induced to differentiate, followed by transfection with *Cpeb4* siRNA for 2 days. The myotubes were then subjected to immunostaining for MHC to assess myotube atrophy. *Cpeb4* knockdown resulted in significantly less MHC-positive areas and smaller myotube diameters, compared with control siRNA treatment (both *p* < 0.005; Figure 6F). To examine whether loss of *Cpeb4* expression is directly related to E3 ligases, we assessed the expression levels of Atrogin-1 (*Fbxo32*) and Murf-1 (*Trim63*) proteins by western blot analysis. Both Atrogin-1 and Murf-1 levels were significantly higher in myotubes with *Cpeb4* knockdown than in those in control cells (both *p* < 0.05; Figure 6G). These findings suggest that *Cpeb4* is key in mediating muscle atrophy via E3 ligases.

To confirm these findings were replicated in aging mice, *Cpeb* expression was measured in 4-, 19-, and 27-month-old animals. *Cpeb4*, but not *Cpeb1–3*, mRNA levels, were significantly lower in the skeletal muscle of 27-month-old mice compared with 4- and 19-month-old mice (*p* < 0.01; Figure 6H). The results suggest a potential role for Cpeb4 in aging-related muscle atrophy based on the observed lower expression of *Cpeb4* in old mice, but further studies are needed to confirm its exact contribution.

## DISCUSSION

A significant goal of the aging field is to develop interventions that both increase lifespan and improve health and quality of life. Among various regeneration strategies, physical exercise and CR promote the function of many tissues in the body and extend the lifespan. However, little is known about the mechanisms by which exercise and CR improve muscle function. In this study, we sought to identify genes in muscle regulated by both exercise and CR. We discovered seven such genes by analyzing expression profiles of GEO datasets obtained from muscle tissue of young men post-exercise and calorie-restricted primates: *ADAMTS1*, *CPEB4*, *EGR2*, *IRS2*, *NR4A1*, *PYGO1*, and *ZBTB43.* In addition, we confirmed the expression of these genes in human muscle tissue, and found that expression of all seven genes increased during myogenic differentiation. Finally, by silencing these genes in C2C12 myoblasts, we found that they were all involved in regulating myogenesis and mitochondrial respiration, but had no effect on autophagy and insulin signaling, all of which are known to be altered by CR and exercise.

Firstly, we discovered that IRS2 plays a key role in myogenesis. IRS proteins mediate IR/IGF-1R signaling by acting as protein scaffolding to form signaling complexes [19]. IRS1 and IRS2 are ubiquitously expressed and have distinct roles within the same tissue [20–22]. In muscle cells, the roles of IRS1 and IRS2 in the mitogenic and metabolic signals of AKT, ERK, and p38MAPK are different. IRS1 controls actin remodeling, AKT1 and AKT2 phosphorylation, and GLUT4 translocation and glucose uptake; IRS2 controls AKT2, activation, while contributing little to other processes [23]. Our findings show that although *Irs2* silencing had no effect on AKT phosphorylation or glucose uptake in C2C12 myotubes, it impaired myotube differentiation and mitochondrial respiration. However, the function of IRS proteins is regulated by diverse factors including free fatty acids, cytokines, angiotensin II, endothelin-1, amino acids, and cellular stress [24]. Thus, exercise and calorie restriction-induced IRS2 upregulation may impact muscle cell function via a previously unexplored pathway, highlighting a potential new mechanism for the beneficial effects of these interventions.

In addition, we found that exercise and CR were associated with elevated *NR4A1* expression, a crucial gene in myogenesis. NR4A1 is a nuclear receptor family of intracellular transcription factors, and is linked with a wide range of cellular activities, including proliferation, differentiation, apoptosis, metabolism, and inflammation [25]. In line with our findings, Xie and colleagues reported an upregulation of *Nr4a1* during myogenesis. They demonstrated that C2C12 myoblast differentiation is accelerated by *Nr4a1* overexpression, while differentiation is inhibited by *Nr4a1* ablation [26]. In addition to myogenesis, we found that *Nr4a1* silencing led to a reduction in maximum respiration, which indicates mitochondrial dysfunction in *Nr4a1*-silenced C2C12 myotubes. Consistent with these results, *Nr4a1* silencing also downregulated the expression of genes involved in mitochondrial oxidative phosphorylation. Similar results were obtained in bone marrow-derived macrophages, where lower basal, ATP synthase-coupled, and peak oxygen consumption rates were observed in *Nr4a1*-deficient bone marrow-derived macrophages than in control cells [27]. Conversely, *Nr4a1*-deficient T cells had higher basal and maximum respiration levels than control cells [28]. These findings indicate that NR4A1 might act differently depending on the tissue. In addition, *Nr4a1* gene expression was lower in diabetic and insulin-resistant rats’ skeletal muscle than in control animals [29]. Taken together, results suggest that *NR4A1* upregulation by exercise and CR may be involved in the enhancement of skeletal muscle function.

Finally, we identified *CPEB4* as a gene upregulated by CR and physical activity, and found that CPEB4 may control muscle atrophy. The RNA-binding protein CPEB, also known as cytoplasmic polyadenylation element binding protein, regulates the length of poly(A) tails, translation, and mRNA transport [30]. Target RNA is typically activated by CPEB for translation, but it can alternatively operate as a repressor [31]. Among CPEB isoforms, CPEB1 is essential for activation of skeletal muscle stem cells. CPEB1 controls stem cell activation and muscle regeneration by attaching to CPEs in the 3′ untranslated regions of Myod1 [32]. Although CPEB4 is linked with abnormal post-transcriptional reprogramming in diseases such as cancer, liver disease, obesity, and neurodegenerative disorders [33–37], there is currently no evidence that CPEB4 serves any function in muscle. However, we found upregulation of CPEB4 after exercise and CR, as well as downregulation of *Cpeb4* expression in the muscle of aging mice. In addition, our RNA-sequencing analysis of C2C12 cells showed that *Cpeb4* knockdown altered the expression of genes linked with myotube differentiation and atrophy; however, functionally, *Cpeb4* knockdown induced myotube atrophy but had little effect on myogenesis. Although CPEBs are differentially expressed in somatic tissues, multiple CPEBs can coexist in a single cell and even co-regulate overlapping populations of transcripts. Thus, it is possible that the targets of CPEB4 and CPEB1 may overlap. Nonetheless, the function of CPEB4 and its target mRNA in skeletal muscle requires verification in future investigations.

Overall, this study provides novel insights into the molecular basis of the beneficial effects of exercise and calorie restriction on skeletal muscle function, and identifies potential targets for future research in the field of aging. However, our study has several limitations. Firstly, we only examined a limited number of genes involved in myogenesis, mitochondrial respiration, autophagy, and insulin signaling. There may be other genes or pathways involved in the effects of exercise and CR that were not examined in this study. Secondly, we only used siRNA to transiently knock down gene expression, which has limitations in terms of duration and specificity. Future studies using more advanced gene editing techniques or viral vectors to regulate gene expression may provide more definitive results. Lastly, our study was limited to analysis of gene expression in muscle tissue. Further studies examining gene expression in other tissues and at different time points may provide a more comprehensive understanding of the effects of exercise and CR on gene expression.

## MATERIALS AND METHODS

### Differential gene expression analysis

RNA-sequencing libraries were prepared using TruSeq Stranded mRNA LT Sample Prep Kit and 101 bp paired-end sequencing was carried out using the Illumina NovaSeq 6000. Alignment to the reference genome (Mus_musculus.GRCm38.dna.primary_assembly) was conducted using STAR version 2.7.3a with the parameters -- outSAMunmapped Within -- outSAMattributes Standard -- twopassMode Basic -- limitOutSJcollapsed 1000000 -- limitSjdbInsertNsj 1000000 -- outFilterMultimapNmax 100 -- outFilterMismatchNmax 33 -- outFilterMismatchNoverLmax 0.3 -- seedSearchStartLmax 12 -- alignSJoverhangMin 15 -- alignEndsType Local -- outFilterMatchNminOverLread 0 -- outFilterScoreMinOverLread 0.3 -- winAnchorMultimapNmax 50 -- alignSJDBoverhangMin 3 -- outFilterType BySJout. The gene count matrix was obtained by featureCounts version 2.0.1 using Mus_musculus.GRCm38.100 as a gene model. For identifying differentially expressed genes, DESeq2 version 1.36.0 was used. The Wald test was used to test for significance. DEGs were defined as having an FDR adjusted *p*-value < 0.05 and absolute fold change ≥1.5. Gene set over-representation analysis was conducted using clusterProfiler version 4.4.4 and genekitr version 1.0.8.

### Cell culture

C2C12 mouse muscle cell lines were purchased from American Type Culture Collection (ATCC, #CRL-1772) and maintained in Dulbecco’s modified Eagle’s medium (DMEM) containing 10% fetal bovine serum (Gibco), 100 IU/mL penicillin, and 100 μg/mL streptomycin. Cells were incubated at 37°C in a humidified atmosphere of 5% CO_2_. Upon 70% confluence, C2C12 cells were harvested using 0.25% trypsin and 0.1% EDTA (Gibco) in PBS. To induced differentiation, C2C12 reached about 80% confluence, and the medium was changed to differentiation medium (DM), consisting in DMEM containing 2% fetal bovine serum, penicillin and streptomycin. The differentiation medium was switched daily until myotube were fully differentiated. For autophagy induction, myotube cells were rinsed with phosphate-buffered saline (PBS) and grown for 6 h in Earle’s Balanced Salt Solution (EBSS) (Sigma-Aldrich). To measure the insulin signaling pathway, myotube cells were incubated for 4 h in serum-free DMEM and then treated with insulin (100 ng/mL, Sigma-Aldrich). siRNA targeting selected genes or control, non-targeting siRNA were transfected into C2C12 myoblasts for 24 h using esiRNA (Sigma-Aldrich). C2C12 myoblasts were transfected using 2.5 ng of each esiRNA using RNAiMAX (Invitrogen), according to the manufacturer’s instructions.

### Mice

Mouse strains used in experiments were in the C57Bl/6J background. Young mice (4-month-old) and aged mice (19- and 27-month-old) were purchased from the Laboratory Animal Resource Center (KRIBB). Mice were housed at 22–24°C in a cage on a 12-hour light, 12-hour dark cycle with free access to food and water. Mice were sacrificed, and isolated tibialis anterior muscle tissue was used for further analysis.

### Gene expression studies

Total RNA was isolated from C2C12 myoblasts and myotubes using RiboEx reagent (GeneAll Biotechnology Co., South Korea). cDNA was synthesized by iScript cDNA synthesis Kit (Bio-Rad). qRT-PCR analysis was performed using StepOnePlus (Applied Biosystems) with a 20 μL final reaction mixture containing cDNA, primers, and SYBR Master Mix (Applied Biosystems). The cycling conditions were as follows: preincubation at 95°C for 10 min, 40 cycles of 15 s at 95°C, and 1 min at 60°C. Experiments were performed in triplicate for each sample. Results were normalized to either *Gapdh* or *36b4*, and fold change was calculated using the 2^−ΔΔCt^ method. qPCR was used to measure the mRNA expression levels of *Fbxo32, Trim63, Ndufs1, Ndufs2, Ndufs8, Sdh, Atp5b, Tfam, Ppargc1a, Acadm, Acca2, Acsl1,* and *Acox1*. The sequences of the primers used in this study are provided in Supplementary Table 1.

### Immunoblotting analysis

For total cell extracts, cells were lysed using RIPA buffer containing 1 mM AEBSF, 0.1 mM Na_3_VO_4_, 1 mM NaF, and 5 mg/mL aprotinin (Sigma-Aldrich) for 20 min. Protein concentration was determined using a BCA (bicinchoninic acid) assay kit and Bradford assay (Thermo Fisher). Samples were denatured by heat at 95°C for 5 min. Protein samples were loaded on SDS-PAGE and transferred to a PVDF and NC membrane. The membrane was blocked for 1 h at RT with nonfat skimmed milk. Primary antibodies were incubated at 4°C overnight. All primary antibodies were used at a 1:1000 dilution. Membranes were incubated with HRP-conjugated secondary antibodies at RT for 1 h. Images were detected using the ChemiDoc (Thermo Fisher, iBright CL1500) and quantification was performed using ImageJ software. The information regarding the antibody is available in the Supplementary Table 2.

### Immunocytochemistry

C2C12 cells were passaged, plated into 24-well culture dishes, and differentiated into myotubes. Myotubes were rinsed with PBS and fixed in 4% paraformaldehyde for 15 min at RT. Myotubes were permeabilized in 0.25% Triton X-100 in PBS for 10 min. Then, myotubes were blocked with 1% BSA containing 0.1% Tween20 in PBS for 30 min at RT and incubated with a primary MYH antibody (1:200, sc-376157) at 4°C overnight. After washing, myotubes were incubated with Alexa Fluor 488 (1:500, #A-21121) for 1 h at RT in the dark. Myotubes were then incubated in Vectashield with DAPI mounting medium (VECTOR Laboratories). Images were analyzed using the Nikon Eclipse Ti-U inverted microscope and Nikon DS-Ri2 camera. The short-axis diameters of myotube were analyzed using NIS-Elements software. Quantification of myosin-positive (Myh+) areas was performed using ImageJ software.

### Glucose uptake

Glucose uptake was measured in C2C12 myotubes transfected with esiRNA for each target gene using a glucose uptake assay kit from Abcam (#ab136955) according to manufacturer’s instructions. C2C12 myotubes were differentiated using 2% FBS DMEM and were transfected 1 day before the assay was performed.

### Oxygen consumption rate (OCR)

The OCR of C2C12 myotubes was measured in 96-well plates using a XF96 Extracellular Flux Analyzer and the XF Cell Mito Stress Kit (Seahorse Bioscience), following the manufacturer’s protocol. C2C12 myotube cells were plated in a 96-well cell culture plate and subsequently transfected with siRNAs for 24 h. Cells were washed and pre-incubated in XF DMEM medium (pH 7.4) for 1 h before the assay. The Mito Stress Tests were performed following the manufacturer’s protocol. Oligomycin (1 μM), FCCP (2 μM), and rotenone and antimycin A (0.5 μM each) were added as indicated.

### Quantification and statistical analysis

All data here are expressed as the mean ± standard error of the mean (SEM). Statistical comparisons were analyzed using a student’s *t*-test. Statistically significant differences are denoted as ^*^p < 0.05, ^**^p < 0.01, ^***^p < 0.005. Data analysis was performed using GraphPad Prism version 9.0.2 (GraphPad Software, San Diego, CA, USA).

## Supplementary Materials

Supplementary Figures

Supplementary Tables
