# Supplementary material for: Identification of novel genes associated with exercise and calorie restriction effects in skeletal muscle
Source: Aging (Albany NY). 2023 Jun 12;15(11):4667–84. doi: 10.18632/aging.204793 (PMC10292903; doi:10.18632/aging.204793)
Supplement: Supplementary Figures [file aging-15-204793-s001.pdf]

SUPPLEMENTARY FIGURES

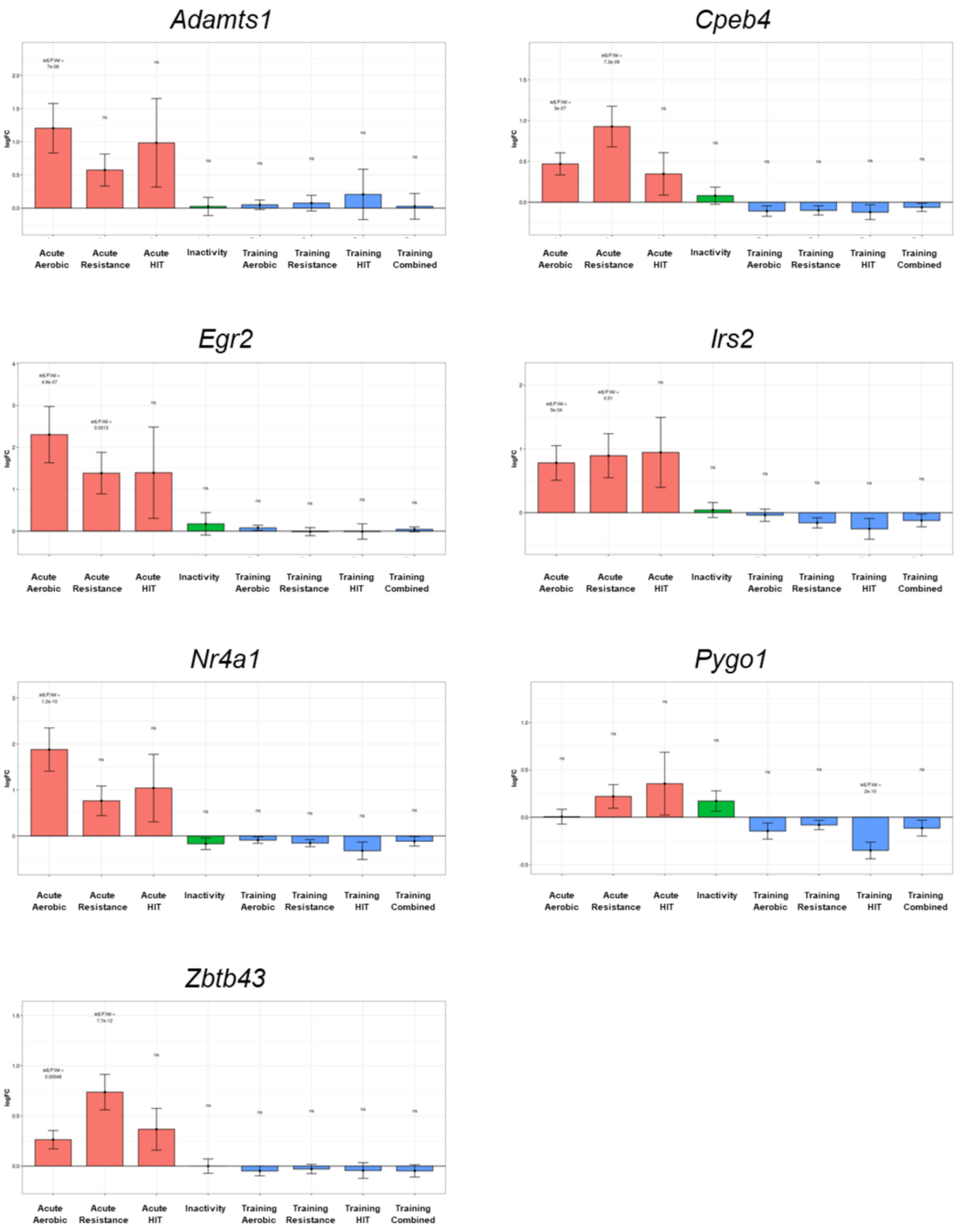

Supplementary Figure 1. Meta-analysis overview of selected genes expression in response to acute aerobic, acute resistance, training aerobic and training resistance exercise (Pillon et al., 2020). The analysis offers annotations of each study based on the individuals collected skeletal muscle type, sex, age, weight, fitness, and metabolic status. Muscle biopsies were collected at 0 h up to 96 h after physical activity.

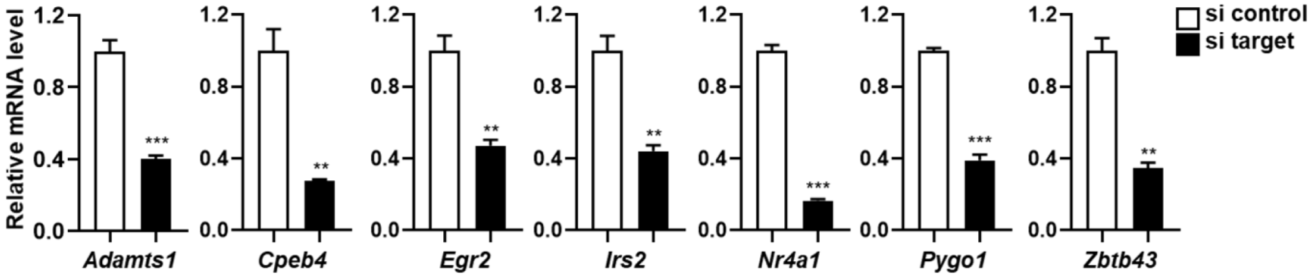

Supplementary Figure 2. Knockdown efficiency of target genes in C2C12 myoblast. q-PCR showing the relative expression of target gene mRNA in C2C12 myoblast was significantly decreased compared with each control cells. All data are represented as mean ± SEM. Statistically significant differences are produced as $^{*}p < 0.05$, $^{**}p < 0.01$, $^{***}p < 0.005$.
