# Supplementary material for: Identification of novel genes associated with exercise and calorie restriction effects in skeletal muscle
Source: Aging (Albany NY). 2023 Jun 12;15(11):4667–84. doi: 10.18632/aging.204793 (PMC10292903; doi:10.18632/aging.204793)
Supplement: Supplementary Tables [file aging-15-204793-s002.pdf]

SUPPLEMENTARY TABLES

Supplementary Table 1. List of primers.

Gene	Forward	Reverse
Acadm	TTG AGT TGA CGG AAC AGC AG	CCC CAA AGA ATT TGC TTC AA
Acca2	CTG ACG TAT ACT GAA CTG GTG TTG GAT G	TTT CCA GGC TAC CAT GCC AAT CTC
Acox1	ACT ACC TGG ACA GCC AAT GC	CCC GAC TGA ACC TGG TCA TA
Acsl1	CCA GAG GGG GAT TCA GGT GTC AAA T	CTT TGT TCA CTA TGT AGG TGA TGG CGT C
Adamt1	GAA GGC AAA CGA GTC CGC TAC A	TTG GGT GTC CAC TCT ACA GTG G
Atp5b	TCCTGCCAGAGACTATGCG	GATGACTGCCACGATTCGC
Cpeb1	GAC TCA GAC ACG AGT GGC TTC A	ACG CCC ATC TTT AGA GGG TCT C
Cpeb2	GAG ATC ACT GCC AGC TTC CGA A	CAA TGA GTG CCT GGA CTG AGC T
Cpeb3	CGG ACC GAT AAT GGT AAC AAT CTG	CAG AGG CTC ATG GTC AGT CCT T
Cpeb4	TCA GCT CCA GAA GTA TGC TCG C	GAG TGC ATG TCA AAC GTC CTG G
Egr2	CCT TTG ACC AGA TGA ACG GAG TG	CTG GTT TCT AGG TGC AGA GAT GG
Fbxo32	ACA AGG GAA GTA CGA AGG AGC G	GGC AGT CGA GAA GTC CAG TC
Irs2	CCA GTA AAC GGA GGT GGC TAC A	CCA TAG ACA GCT TGG AGC CAC A
Ndufs1	AGG ATA TGT TCG CAC AAC TGG	TCA TGG TAA CAG AAT CGA GGG A
Ndufs2	GCA AGG AAT TTG CAT AAG ACA GC	TAG CCA TCC ATT CTG CCT TTG
Ndufs8	GGC ACG TGT TCC CTT CCT AC	CCG CTC CAG GTA CAG ATT ATT GT
Nr4a1	GTG CAG TCT GTG GTG ACA ATG C	CAG GCA GAT GTA CTT GGC GCT T
Ppargc1a	GGA CAT GTG CAG CCA AGA CTC	CAC TTC AAT CCA CCC AGA AAG CT
Pygo1	TGG ACT GGA TGG GTT AGG AGG G	TGG TCG GAG TTT GGA TTC GGT G
Sdh	GGA CCT ATG GTG TTG GAT GC	GTG TGC ACG CCA GAG TAT TG
Tfam	ATT CCG AAG TGT TTT TCC AGC A	TCT GAA AGT TTT GCA TCT GGG T
Trim63	GTG TGA GGT GCC TAC TTG CTC	GCT CAG TCT TCT GTC CTT GGA
Zbtb43	AGC ATC ATG GCT CAT AGG CGC T	TCA GTG ACC TGG TGC TCA TCG T

Supplementary Table 2. List of antibodies.

Primary antibody	Company	Cat#
MHC	SCBT	SC-376157
Myogenin	SCBT	SC-52903
HSP90	BD	610418
P62	PROGEN	GP62-C
LC3-III	CST	12741S
P-AKT (S473)	CST	9271L
P-AKT (T308)	CST	9275S
AKT	SCBT	SC-1618
